# Psychological Well-Being Increment as Post-Traumatic Growth in Women with Breast Cancer: A Controlled Comparison Design Using Propensity Score Matching

**DOI:** 10.3390/healthcare10081388

**Published:** 2022-07-25

**Authors:** Ren-Hau Li, Hsiu-Ling Peng, Ming-Hsin Yeh, Jiunnhorng Lou

**Affiliations:** 1 Department of Psychology, Chung Shan Medical University, Taichung 40201, Taiwan; phl@csmu.edu.tw; 2 Clinical Psychological Room, Chung Shan Medical University Hospital, Taichung 40201, Taiwan; 3 Department of Surgery, Chung Shan Medical University Hospital, Taichung, Taiwan, Institute of Medicine, School of Medicine, Chung Shan Medical University, Taichung 40201, Taiwan; cshy1629@csmu.edu.tw; 4 Department of Nursing, Hsin Sheng College of Medical Care and Management, Taoyuan 325004, Taiwan

**Keywords:** breast cancer, propensity score, factorial invariance tests, psychological well-being, post-traumatic growth, latent variables

## Abstract

The aim of this study was to confirm post-traumatic growth with respect to the psychological well-being of women with breast cancer compared to women without disease. Propensity score was used to match the two groups according to age, religious beliefs, education level, monthly income, and marital status. A psychological well-being scale with six factors was used, including positive relations with others (PR), autonomy (AU), environmental mastery (EM), personal growth (PG), purpose in life (PL), and self-acceptance (SA). A total 178 women with vs. 178 women without breast cancer were compared by matching with propensity scores, using factorial invariance tests to reduce measurement errors. The results showed that women with breast cancer had significantly higher psychological well-being for all the six factors (Δ*χ*^2^ = 37.37, *p* < 0.001) and higher variability in terms of PR, AU, and PL than women without breast cancer (Δ*χ*^2^ = 45.94, *p* < 0.001). Furthermore, women with breast cancer exhibited a significantly higher association between PG and PL and a significantly lower association between PG and EM than women without breast cancer (Δ*χ*^2^ = 44.49, *p* < 0.001). This implies that psychological well-being could assess broader and more subtle post-traumatic growth in women with breast cancer and that growth was more associated with internal life value than with external environmental control.

## 1. Introduction

Post-traumatic growth, a positive phenomenon that usually occurs after stressful events, is a type of transformational change that occurs as a positive response to challenges to one’s core beliefs following genuinely traumatic events [1]. However, stressful events may also result in severely negative outcomes in persons with cognitive, emotional, or behavioural distress or injuries, called traumas, such as receiving a diagnosis of breast cancer. Trauma can be briefly defined as ‘a life-altering event that is seismic enough to impact one’s assumptive world, prioritising one’s subjective response to significantly challenging events’ [2]. Individuals who have undergone trauma may exhibit positive and/or negative responses. Some negative responses may lead to psychological disease, known as post-traumatic stress disorder, as per the Diagnostic and Statistical Manual of Mental Disorders: Fifth Edition (DSM-V). In contrast, some positive responses or benefits may result in growth, including strengthening of individuals, families, and communities; discovery of previously unrecognised abilities and talents; and tightening of relationships and solidarity [3]. Post-traumatic growth is also viewed as a positive psychological change experienced due to struggles with highly challenging life circumstances and is usually negatively associated with depression, anxiety, and stress [4,5], which are also associated with poor breast cancer prognosis [6].

Breast cancer, the most frequent malignancy in women worldwide, has a curability rate of 70–80% in patients with early-stage, non-metastatic disease. However, advanced breast cancer with distant organ metastases is considered incurable [7]. Breast cancer is usually viewed as a trauma [8] associated with anxiety, depression, suicide, and neurocognitive and sexual dysfunctions, according to a systematic review [9]. Furthermore, it leads to psychological and physiological distress, such as stress, demoralisation, and sleeping disturbances [10]. In 2020, breast cancer surpassed lung cancer as the most commonly diagnosed cancer, with an estimated 2.3 million new cases (11.7%) worldwide. Furthermore, it is the leading cause of cancer deaths among women [11]. In Taiwan, breast cancer is the most rapidly growing cancer and the leading cause of cancer deaths among women, with over 10,000 women diagnosed and more than 2000 women dying from breast cancer annually [12]. Although breast cancer is usually prevalent in middle-aged and older women, its prevalence has been increasing among youth in Taiwan [13,14].

Positive psychology, which encourages facing diseases with positive attitudes, has been advocated in the last 20 years [15], and researchers have started to consider the positive benefits of traumas, such as quality of life, post-traumatic growth, social support, and well-being [16,17]. Approximately 30–70% of participants in various studies reported benefits from trauma [18], and 97% of women with breast cancer experienced post-traumatic growth [19]. However, most studies on positive psychology-related variables of breast cancer did not confirm the post-traumatic growth phenomena via a control group design or even by matching participants via confounding or sociodemographic variables, much less by comparisons among latent variables to avoid measurement errors.

Only one study compared post-traumatic growth in women with and without breast cancer using the matching method [20]. A study including 774 women with breast cancer and 666 randomly sampled women without breast cancer adjusted for age and education in a regression analysis found that although the group with breast cancer had significantly higher post-traumatic growth scores in two of the five subscales, there were no significant differences in terms of the total post-traumatic growth scale [21]. Cordova et al. compared 70 breast cancer survivors to 70 age- and education-matched healthy women. Their findings indicated that the group with breast cancer had higher post-traumatic growth scores; however, there were no significant differences in depression and psychological well-being [20]. The lack of a significant difference in depression conflicted with previous systematic reviews and meta-analyses [9,22]. In addition, it is worth noting that no significant difference was observed in psychological well-being was. Because only one study used a matching method [20], we found it necessary to implement a comparative design with advanced methods.

In the present research, in to reach achieve more equivalent groups in random assignment design, we used propensity score to match two groups of women with/without breast cancer based on five sociodemographic variables—age, presence or absence of religious belief, educational level, monthly income level, and marital status—which are often mentioned in the literature [9,20,23,24]. The advantage of the propensity score method is that it reduces selection bias in observational studies while simultaneously considering many confounding variables in matching [25]. In addition, we operationally defined the difference between the two groups on the psychological well-being scale as post-traumatic growth and discussed their differences. Comparisons were executed for latent variables via a factorial invariance test through confirmatory factor analysis in structural equation modelling [26]. The primary aim of the study was to use a control-group setting with advanced statistical control and techniques to confirm the existence of post-traumatic growth in women with breast cancer. The secondary aim was to use the Psychological Well-Being Scale to identify post-traumatic growth and its related phenomena. We assumed that the psychological well-being increments (differences between groups) would lead to the discovery of some extent of post-traumatic growth for women with breast cancer.

## 2. Materials and Methods

### 2.1. Procedures

A cross-sectional survey was mainly conducted in Taiwan from August 2013 to September 2015. However, a few participants with breast cancer were recruited from December 2020 to April 2021 before the outbreak of COVID-19 in Taiwan. The questionnaire included questions about age, religious beliefs, education level, monthly income, marital status, and a psychological well-being scale. This study was approved by the Institutional Review Board of Chung Shan Medical University Hospital (CSMUH Nos. CS-13203 and CS1-20158). We adopted a convenience sampling method to recruit women with or without breast cancer from the central area of Taiwan. The participants were informed of the research contents, their rights, and privacy protection and were administered the scale by trained researchers and assistants after they agreed to participate in the study. Collected data were checked for the purpose of matching similar sociodemographic variables to guarantee post-traumatic growth mainly from events associated with breast cancer. Propensity score matching was executed using SPSS statistical software version 23 to find qualified participants for later analysis and factorial invariance tests.

### 2.2. Participants

A total of 351 women with breast cancer agreed to participate. We also recruited 226 women without breast cancer, who were included in the control group. Because the two sample groups had different distributions of sociodemographic variables, we matched participants according to their age, religious beliefs, educational level, monthly income, and marital status using propensity scores. We set a caliper of 0.05 in terms of propensity scores to select 178 women with and 178 women without breast cancer. The former group had a mean age of 57.56 years and standard deviation of 6.70, with an age range of 39.0 to 76.0. The latter had a mean age of 57.78 years and standard deviation of 6.61, with an age range of 42.7 to 77.4. Among 178 women with breast cancer, 88.2% had undergone surgery, 65.7% had received chemotherapy, 58.4% had received radiation therapy, and 41.6% had received hormone therapy. In addition, 10.2% had received at least one of the above-mentioned treatments, and 83.1% had received at least two of the above-mentioned treatments; however, 6.7% had not received any of the above-mentioned treatments.

### 2.3. Instrument

We adopted a brief 18-item psychological well-being scale from Li’s version based on Ryff’s efforts, which was reported to have validity and reliability in middle-aged and older samples [27]. The scale included six factors/subscales: positive relations with others (PR), autonomy (AU), environmental mastery (EM), personal growth (PG), purpose in life (PL), and self-acceptance (SA). Each subscale has three items, and each item was rated on a 6-point Likert scale. The higher the scale score, the higher the level of psychological well-being. The scale had Cronbach’s reliability coefficient alpha of 0.92 and 0.82–0.90 for each subscale in the present study. Confirmatory factor analysis of the scale showed that chi-square (χ^2^) = 393.97, degree of freedom (*df*) = 120, *p* < 0.001, χ^2^/*df* = 3.28, comparative fit index (CFI) = 0.98, non-normed fit index (NNFI) = 0.97, goodness-of-fit index (GFI) = 0.89, standardized root mean squared residual (SRMR) = 0.049, and root mean error of approximation (RMSEA) = 0.080, which indicated an acceptable model fit. The factor loadings ranged from 0.72 to 0.93, with a mean factor loading of 0.82.

### 2.4. Statistical Analysis

The propensity score method is a statistical technique used to reduce selection bias in observational study samples to equate the distribution of confounding covariates, such as age, religious beliefs, educational level, marital status, and monthly income level, between the two groups. Based on logistic regression, the propensity score represents the probability that each participant was assigned to a particular group, given a set of confounding observed covariates [25]. The matching processes involved pairing participants in the two groups based on similar propensity scores (probability difference within 0.05), which resulted in two groups with similar distributions of confounding covariates, as shown in the Results section. The skewness coefficients of the total score on the psychological well-being scale and its six subscale scores ranged from −0.268 to 0.237, and the kurtosis coefficients ranged from −0.586 to 0.661, i.e., close to zero, satisfying normality in the sample of the 356 matched participants.

Next, factorial invariance tests were used to confirm that differences in the observed scores between groups reflected true differences in psychological constructs and that they were free from the interference of measurement errors [26]. The tests were used to verify that the measurement models for groups of women with and without breast cancer had full or partial metric and scalar invariance, which was necessary to guarantee the next feasible comparisons between the parameter estimates in the structural models of the two groups. The testing processes were executed using chi-squared difference (Δ*χ*^2^) tests between the nested models. The metric invariance and scalar invariance tests were used to verify the extent of equality for factor loadings and intercepts in the process of confirmatory factor analysis, respectively. Metric invariance ensured that the unit of the scale was consistent, and scalar invariance ensured that the origin of scale was consistent between the two groups. Whereas the two groups had the same unit and origin of the scale, their comparisons in each factor of psychological well-being were meaningful. Partial invariances in the measurement models were also capable of guaranteeing that comparisons in the structural model between groups were feasible [28,29]. We used Lisrel 8.8 statistical software to conduct factorial invariance tests.

## 3. Results

### 3.1. Sociodemographic Information after Matching

A *t*-test showed no significant difference in age (*t* = −0.315, *p* = 0.753). The other sociodemographic variables of the matched samples showed consistent distributions between the two groups, according to chi-squared tests, as shown in Table 1. The non-significant results for the sociodemographic variables indicated that matching through propensity scores was successful.

### 3.2. Factorial Invariance Tests

Table 2 shows the steps of the factorial invariance tests involving measurement and structural models. In the measurement model, the configural model invariance test checked that the two groups (with and without breast cancer) had the same number of factors with acceptable model-fit indices, such as RMSEA = 0.092 (<0.10), CFI = 0.969 (>0.090), and SRMR = 0.058 and 0.057 (<0.08). The full metric invariance test confirmed the same factor loadings between the two groups, with a non-significant chi-squared difference (Δ*χ*^2^) of 17.90. Although full scalar invariance was not satisfied due to a Δ*χ*^2^ of 32.24 with *df* = 12, reaching a significance level of *p* < 0.001, partial scalar invariance was satisfied, with a non-significant Δ*χ*^2^ of 15.83 and *df* = 10, and only two intercepts were freely estimated. The next test, which involved invariance of measurement errors, also indicated that partial invariance of error variances was satisfied (Δ*χ*^2^ = 18.89, *df* = 12); however, it was not important for parameter comparisons in the structure model [28]. Therefore, the next invariance tests, which involved factor covariances, variances, and means in the structural model, were based on model D instead of model F to avoid excessive distortion in the next invariance tests [30].

Because the unit and origin of scale were the same between the two groups for metric invariance and scalar invariance, partial invariance tests of factor covariances, variances, and means provided information regarding the true differences in terms of psychological well-being between women with and without breast cancer. As shown in Table 3, we found that the factor means (latent means) were differed across six factors between the two groups (Δ*χ*^2^ = 37.37, *df* = 6, *p* < 0.001). The group of women with breast cancer consistently had significantly higher means than the group of women without breast cancer, such as higher means for positive relations with others (PR: 3.38 vs. 3.13), autonomy (AU: 3.18 vs. 3.04), environmental mastery (EM: 3.78 vs. 3.43), personal growth (PG: 4.00 vs. 3.64), purpose in life (PL: 3.74 vs. 3.53), and self-acceptance (SA: 3.46 vs. 3.25).

Table 4 shows the covariances and variances of the six factors for the two groups under a completely standardised common metric solution. Women with breast cancer had significantly larger variances in three of the six factors (Δ*χ*^2^ = 45.94, *df* = 6, *p* < 0.001)—positive relations with others (1.14 vs. 0.86), autonomy (1.38 vs. 0.62), and purpose in life (1.31 vs. 0.69)—than women without breast cancer. In addition, women with breast cancer had a significantly larger correlation coefficient (Δ*χ*^2^ = 44.49, *df* = 15, *p* < 0.001) between personal growth and purpose in life (0.86 vs. 0.61) and a lower correlation coefficient between personal growth and environmental mastery (0.48 vs. 0.68) than women without breast cancer.

## 4. Discussion

In the present study, using the propensity score method, we found that the matched samples had similar sociodemographic distributions between women with and without breast cancer. The research results support the hypothesis that women with breast cancer have higher levels of psychological well-being than women without breast cancer across six factors.

Benefits or positive changes that arise from trauma can be viewed as adversarial growth, stress-related growth, benefit finding, and positive psychological changes [3,31]. These gains in the aftermath of suffering are known as post-traumatic growth and were developed to form a post-traumatic growth inventory [32]. This inventory includes 21 items across five factors: new possibilities, relating to others, personal strength, spiritual change, and appreciation of life. In terms of psychological well-being, it is also a type of eudaimonism approach, such as the post-traumatic growth inventory [33], as opposed to hedonism-like well-being, i.e., it originates from growth as a result of a struggle with suffering, not simply happiness resulting from a sensory feeling. Therefore, psychological well-being generally refers to broader growth experiences of persons facing problems or challenges, from light, trivial problems to serious, stressful events—not just trauma. We believe that using the psychological well-being scale could help to evaluate broader overall growth in women with breast cancer. Furthermore, we believe that the post-traumatic growth inventory measures deep awareness of changes involving trauma.

Our findings that all six factors differed significantly between the two groups are roughly consistent with those reported by Brix et al. [21], who found statistically significant post-traumatic growth in two aspects—appreciation of life and relating to others— but not in new possibilities, personal strength, spiritual change, or overall post-traumatic growth. Their aspect ‘appreciation of life’ was similar to our factor ‘purpose in life’, and their aspect ‘relating to others’ was similar to our factor ‘positive relations with others.’ Therefore, Brix et al. did not characterise post-traumatic growth in the other three aspects or according to an overall scale. As they indicated, ‘severity of breast cancer played a role in the development of post-traumatic growth’, not just having breast cancer or not. Moreover, given advancements in medical technology, breast cancer is no longer considered a serious or incurable disease. Currently, it is a relatively mild cancer, with a 91% 5-year survival rate after diagnosis, making it the 5th most survivable of 23 cancers [34]. Therefore, the severity of breast cancer could be interpreted as a subjective feeling and not merely a diagnosis with respect to the stages of cancer or the corresponding treatments. In the present study, broadly speaking, the various types of growth among women with breast cancer were significant compared to those experienced by women without breast cancer in terms of psychological well-being. This could imply that although some women with breast cancer did not experience post-traumatic growth, they actually grew in terms of psychological well-being. Therefore, we viewed the difference (increment) of psychological well-being between the two groups as a kind of post-traumatic growth.

Cordova et al. [20] reported significantly higher levels of post-traumatic growth in relation to others, appreciation of life, and spiritual change, which was similar to the results reported by Brix et al. However, they did not find significant differences in terms of psychological well-being, unlike our results. Cordova et al. used only three of the six subscales of the psychological well-being scale, namely personal growth, purpose in life, and self-acceptance, and reported no significant difference between the groups. Moreover, they recruited participants who had undergone surgery, chemotherapy, and radiotherapy, which ensured the development of post-traumatic growth [35]. However, we found that three of our six factors had significantly higher variability in women with breast cancer. This may imply that the sample in the study by Cordova et al. was too homogenous and not sufficiently large, which led to low variance and insignificance. This inference was directly supported by comparison of the sizes of standard deviations with those reported by Brix et al. [21] and indirectly supported by the conflicts with a systematic review by Carreira et al. [9] regarding the significance of depression. More evidence is required to confirm this hypothesis.

We also found that the correlation coefficient between personal growth and purpose in life was larger in women with breast cancer than in women without breast cancer. Furthermore, the correlation coefficient between personal growth and environmental mastery was smaller in women with breast cancer than in women without breast cancer. As for the connotations of environmental mastery and purpose in life [36], environmental mastery meant that people had good control of living situations, daily life, and finances, whereas purpose in life meant making positive plans and struggling to fulfil them. However, the former was more subject to many external factors, and the latter was more focused on self-determination. This result may imply that women with breast cancer were able to pursue their internal values to a greater extent than they were able to control the outer environment in association with their personal growth. That is, even if women with breast cancer had significantly higher scores for all six factors, their post-traumatic growth may have been more associated with the achievement of an internal value rather than built-in outer environmental controls. This result is roughly consistent with empirical research on patient-perceived changes in the system of values after cancer diagnosis involving terminal and instrumental values [37].

Other cancer-related background variables, including post-traumatic growth, such as cancer stage, time since diagnosis or operation, and treatment type [4,20,21], could not be considered in the matching, because the control group had no such variables. However, these variables may have affected the research results. They should be controlled experimentally or statistically and should not be simply described or listed as characteristics of the participants. Furthermore, we did not consider matching some related psychological variables, such as personality traits, coping style, and social support. Although the authors of many studies have found that such variables were associated with post-traumatic growth [38,39,40,41], we suggest that personality traits are much more worthy of consideration in future research, as they are essentially innate dispositions and are not easily changed in response to stressful events or trauma.

## 5. Conclusions

To confirm post-traumatic growth in women with breast cancer in an observational study, we used propensity scores to match sociodemographic variables between women with and without breast cancer. In addition, we used a factorial invariance test to reduce measurement errors and found a difference in psychological well-being between the two groups in terms of latent variables. Women with breast cancer had significantly higher levels of psychological well-being than women without breast cancer, including positive relations with others, autonomy, environmental mastery, personal growth, purpose in life, and self-acceptance. These findings confirm women with breast cancer experienced post-traumatic growth to some extent. Women with breast cancer exhibited significantly larger variability than women without breast cancer among the three factors of psychological well-being of positive relations with others, autonomy, and purpose in life. This may imply that psychological well-being can assess broader and more subtle post-traumatic growth. Furthermore, women with breast cancer had a significantly larger associations between personal growth and purpose in life and a significantly lower association between personal growth and environmental mastery than did women without breast cancer. This may imply that post-traumatic growth is more associated with internal life value than with external environmental control.

The main limitation of the present research is that conducting a study like ours using an experimental design with randomization would be unethical; hence, a longitudinal panel study with a control group design is preferable. In addition, indicators of severity of breast cancer, such as time since diagnosis, stage, and treatment type, may have influenced the results of our study; therefore, they should be considered subjective measures of severity as a psychological variable to be controlled in future studies.

## Figures and Tables

**Table 1:** Sociodemographic information of the participants (*n* = 356).

Sociodemographic Variable	*n* (%)		Chi-Square (*χ*^2^)
	Breast Cancer	Normal	
Religious belief			0.11
No	19 (10.7%)	21 (11.8%)	
Yes	159 (89.3%)	157 (88.2%)	
Educational level			2.79
Illiterate	5 (2.8%)	9 (5.1%)	
Elementary school	31 (17.4%)	34 (19.1%)	
Junior high school	26 (14.6%)	19 (10.7%)	
Senior high school or university	110 (61.8%)	112 (62.9%)	
Graduate degree or above	6 (3.4%)	4 (2.2%)	
Monthly income			7.56
Less than TWD 20 thousand	97 (54.5%)	78 (43.8%)	
TWD 20–50 thousand	56 (31.5%)	61 (34.3%)	
TWD 50–80 thousand	14 (7.9%)	29 (16.3%)	
TWD 80 thousand	11 (6.2%)	10 (5.6%)	
Marriage			2.85
Unmarried/single	9 (5.1%)	14 (7.9%)	
Married/cohabited	142 (79.8%)	130 (73.0%)	
Divorced/separated	15 (8.4%)	16 (9.0%)	
Widowed	12 (6.7%)	18 (10.1%)	

**Table 2:** Tests of factorial invariance of the psychological well-being scale between women with and without breast cancer (*n* = 356).

Models	Compared Model	*χ*^2^ (*df*)	RMSEA	CFI	SRMR	Δ*χ*^2^ (Δ*df*)
A. Configural invariance		600.62 (240)	0.092	0.969	0.058/0.057	
B. Full metric invariance	A	618.52 (252)	0.091	0.968	0.065/0.062	17.90 (12)
C. Full scalar invariance	B	650.76 (264)	0.091	0.966	0.065/0.058	32.24 *** (12)
D. Partial scalar invariance	B	634.35 (262)	0.090	0.967	0.065/0.059	15.83 (10)
E. Full invariance of error variances	D	698.49 (280)	0.092	0.963	0.067/0.063	64.14 *** (18)
F. Partial invariance of error variances	D	653.24 (274)	0.088	0.972	0.066/0.062	18.89 (12)
G. Full invariance of factor variances	D	680.29 (268)	0.093	0.964	0.144/0.211	45.94 *** (6)
H. Partial invariance of factor variances	D	641.67 (265)	0.090	0.967	0.108/0.107	7.32 (3)
I. Full invariance of factor covariances	D	678.84 (277)	0.091	0.964	0.122/0.094	44.49 *** (15)
J. Partial invariance of factor covariances	D	653.42 (275)	0.088	0.966	0.112/0.079	19.07 (13)
K. Full invariance of latent means	G	717.66 (274)	0.096	0.961	0.137/0.193	37.37 *** (6)

*** *p* < 0.001.

**Table 3:** Invariant and noninvariant factor loadings, intercepts, error variances, and mean differences between women with and without breast cancer.

Factors	Items	Factor Loadings	Intercepts	Error Variances	Latent Mean
PR	PR1	0.81	−0.45	0.33	
	PR2	0.77	0.13	0.39	
	PR3	0.79	0.39	0.39	3.38/3.13
AU	AU4	0.73	0.48	0.58/0.36	
	AU5	0.83	−0.32	0.31	
	AU6	0.79	−0.14	0.45/0.29	3.18/3.04
EM	EM7	0.84	−0.22/−0.31	0.28	
	EM8	0.89	0.01	0.22	
	EM9	0.81	0.21/0.31	0.33	3.78/3.43
PG	PG10	0.76	0.28	0.41	
	PG11	0.92	−0.15	0.11/0.20	
	PG12	0.91	−0.15	0.17	4.00/3.64
PL	PL13	0.85	−0.37/−0.24	0.27	
	PL14	0.91	−0.38	0.26/0.13	
	PL15	0.75	0.80/0.66	0.44	3.74/3.53
SA	SA16	0.72	0.22	0.62/0.36	
	SA17	0.91	−0.77	0.15	
	SA18	0.76	0.57	0.53/0.34	3.46/3.25

Note: All estimates are presented in a completely standardised common metric solution. For factor loadings, intercepts, and error variances, non-invariant estimates are presented as a pattern of with/without breast cancer, and invariant estimates are presented as a single value. PR, positive relations with others; AU, autonomy; EM, environmental mastery; PG, personal growth; PL, purpose in life; SA, self-acceptance.

**Table 4:** Interfactor correlation of the six factors of PWB between women with and without breast cancer (*n* = 356).

	PR	AU	EM	PG	PL	SA
PR	1.14/0.86					
AU	0.51	1.38/0.62				
EM	0.64	0.60	1.00			
PG	0.66	0.54	0.48/0.68	1.00		
PL	0.53	0.46	0.50	0.86/0.61	1.31/0.69	
SA	0.66	0.57	0.66	0.66	0.68	1.00

Note: All estimates are presented in a completely standardised common metric solution; hence, some values are higher than 1.00. Non-invariant estimates between groups are presented as a pattern of with/without breast cancer; invariant estimates are presented as a single value. PR, positive relations with others; AU, autonomy; EM, environmental mastery; PG, personal growth; PL, purpose in life; SA, self-acceptance.

## Data Availability

The data presented in this study are available upon request, owing to privacy restrictions.
